# Prolonged delirium misdiagnosed as a mood disorder

**DOI:** 10.1590/1980-57642016dn11-020014

**Published:** 2017

**Authors:** Fei Cao, Haitham Salem, Caesa Nagpal, Antonio L. Teixeira

**Affiliations:** 1 Neuropsychiatry Program, Department of Psychiatry and Behavioral Sciences, McGovern Medical School, The University of Texas Health Science Center at Houston, Houston, Texas, USA; 2 Harris County Psychiatric Center, Department of Psychiatry and Behavioral Sciences, McGovern Medical School, The University of Texas Health Science Center at Houston, Houston, Texas, USA

**Keywords:** delirium, mood disorder, general hospital, *delirium*, transtorno do humor, hospital geral

## Abstract

Delirium can be conceptualized as an acute decline in cognitive function that typically lasts from hours to a few days. Prolonged delirium can also affect patients with multiple predisposing and/or precipitating factors. In clinical practice, prolonged delirium is often unrecognized, and can be misdiagnosed as other psychiatric disorders. We describe a case of a 59-year-old male presenting with behavioral and cognitive symptoms that was first misdiagnosed as a mood disorder in a general hospital setting. After prolonged delirium due to multiple factors was confirmed, the patient was treated accordingly with symptomatic management. He evolved with progressive improvement of his clinical status. Early diagnosis and management of prolonged delirium are important to improve patient prognosis and avoid iatrogenic measures.

## Abstract (pt)

*Delirium* pode ser conceituado como o declínio agudo na função cognitiva que geralmente dura de horas a dias. O delírio prolongado também pode afetar pacientes com múltiplos fatores predisponentes e/ou precipitantes. Na prática clínica, o delírio prolongado é muitas vezes não reconhecido, e pode ser diagnosticado como outros transtornos psiquiátricos. Aqui, descrevemos o caso de um homem de 59 anos apresentando sintomas comportamentais e cognitivos que foi diagnosticado inicialmente com transtorno de humor em um hospital geral. Após ser diagnosticado *delirium* prolongado devido a múltiplos fatores, o paciente foi tratado de acordo, evoluindo com melhora progressiva do seu estado clínico. O diagnóstico e o manejo precoces do *delirium* prolongado são importantes para melhorar o prognóstico do paciente e evitar medidas iatrogênicas.

## INTRODUCTION

Delirium is usually characterized by an acute onset of mental status and cognitive changes.^1^ It can be categorized into hypoactive, hyperactive or mixed types. Hypoactive and mixed types together account for approximately 80% of delirium cases.^2^ Generally, delirium is reversible within a short time period (from hours to days), and full recovery is common once the underlying cause(s) has/have been recognized and eliminated.^3^

Prolonged delirium can occur in patients with multiple predisposing and/or precipitating factors, and has far poorer functional outcome and increased mortality.^4^ Unfortunately, prolonged delirium is often unrecognized or misdiagnosed as other psychiatric conditions in clinical practice, such as dementia, mood disorders, or psychosis.^5,6^ Consequently, attention should be paid to early recognition and diagnosis of delirium in order to limit its persistence and improve patient prognosis.^7^

We present a case of prolonged delirium with multiple brain insults that was misdiagnosed as a mood disorder.

## CASE DESCRIPTION

A 53-year-old Hispanic male was referred to our psychiatric hospital with a diagnosis of "mood disorder with psychosis". At the initial evaluation, he was irritable, endorsing depressive symptoms and reporting auditory and visual hallucinations. The patient had a long-standing history of alcohol use disorder, but no other psychiatric disorder. His past medical history included uncontrolled hypertension and seizures.

He had been referred from a general hospital after a two-week stay. According to the hospital's records, he had been admitted with similar symptoms, i.e. complaints of irritability and depressed mood. Laboratory tests revealed hyponatremia, with sodium level of 121 mmol/L. Fluids were started for hyponatremia management along with thiamine, folate, and multivitamin replacement. Chlordiazepoxide was started for prevention of alcohol withdrawal symptoms. Levetiracetam was also prescribed due to his past history of seizures, where it remained unclear whether these were related to alcohol withdrawal. The patient had no seizure episodes during the hospital stay. After clinical stabilization, including correction of hyponatremia, he had been discharged.

**Figure 1:**
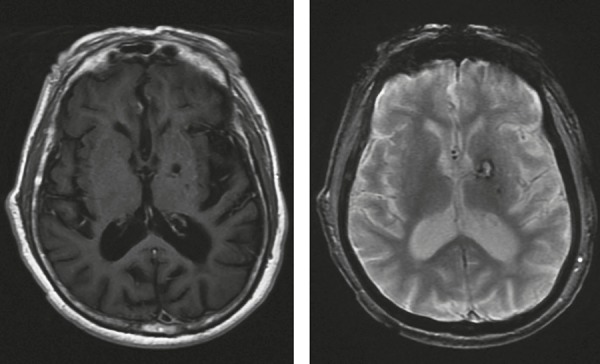
T1 and T2* magnetic resonance imaging sequences depicting old infarct area in the left basal ganglia.

In our psychiatric hospital, it was noted that the patient was easily distracted and had difficulty keeping track of what he was talking about. He was disoriented for time and place with impaired attention/concentration (failure in serial 7's) and long-term memory (recall of list of words). Neurological examination revealed that the patient had right-hand dystonia and very mild right hemiparesis (Figure 1). No signs of ataxia, gait impairment or ophthalmoparesis (i.e. signs of Wernicke encephalopathy) were observed. Therefore, cranial MRI was ordered, revealing an earlier subcortical stroke (Figure 1). Notably, the patient's behavior worsened at night with greater disorientation and agitation. The results of laboratory exams, including hemogram, iono-gram, glycemia, thyroid, renal and liver functions, folate and B12 levels, were all within normal ranges.

Although the patient was not exhibiting physical signs of alcohol withdrawal (receiving no doses of benzodiazepines during psychiatric admission) and had unremarkable laboratory exams, the diagnosis of prolonged delirium due to multiple factors was established. In addition to his previous medications, the patient was started on risperidone (1 mg at bedtime), and encouraged to follow the structured agenda of the psychiatric ward.

During the ensuing two weeks, the patient's conditions and symptoms gradually improved. Before discharge, he was oriented with regard to time and place. The patient was later discharged to a nursing facility for further recovery and support.

## DISCUSSION

Clinical features of delirium include altered level of consciousness, changes in cognition, and perceptual disturbances.^8^ Delirium is associated with increased mortality, prolonged hospital stay, and long-term neuropsychological deficits. These poor outcomes are not only related to the development of delirium but also associated with its duration.^9^ In general hospital settings, delirium is the most often encountered psychiatric diagnosis with an incidence of up to 82% in ICU,^10^ and is frequently unrecognized or misdiagnosed in up to 70% of older patients.^2^

In our patient, the diagnosis of "mood disorder with psychosis" was given to the patient in the general hospital once the signs of alcohol withdrawal were no longer observed, and hyponatremia had been corrected. However, he evolved with cognitive and behavioral fluctuation, leading to the diagnosis of delirium. It is noteworthy that no signs of infectious conditions, biochemical changes or alcohol withdrawal were evident at the time.

The pathophysiology of delirium is complex. It is widely acknowledged that delirium results from the interplay of multiple predisposing and/or precipitating factors, including medical diseases, medications, drugs, metabolic disorders, nutritional deficiency, acute trauma, infection and impaired physical or functional abilities. In contrast to short-term delirium, the risk factors associated with prolonged delirium, have not yet been fully determined. Our patient had multiple problems, including alcohol use disorder, cerebrovascular disease, seizures, and possibly nutrition deficiency. All these conditions may be regarded as predisposing, but the ultimate cause of delirium is complex to define, especially in the context of prolonged delirium with no clinically evident biochemical change or infection.

Recognition of delirium still relies on individual clinical experience, a high degree of suspicion, and repeated cognitive testing of at-risk individuals.^11^ Moreover, its diagnosis remains an under researched area.^11^ As a result, even though delirium is very common in clinical practice, it remains a "confusing" condition for most health practitioners.^12^

Since prolonged duration poses a greater risk for poor functional outcomes, early recognition and management of delirium is critical.^1^ Proactive strategies to target defined risk factors and/or physiological factors seem vital to prevent and manage prolonged delirium and its relevant consequences. Potential measures include comprehensive assessment of patients, therapeutic environmental modification, standardized protocols for physiological interventions, medical staff education, limiting use of sedating medications (especially benzodiazepines), and perhaps, judicious use of antipsychotics.^13^ Medical staff must have relevant knowledge to identify risk factors and implement preventive strategies. In general hospitals, it is very important to consider the diagnosis of delirium for patients who exhibit sudden changes in mental status, adopting the necessary steps to provide safe and effective medical care. Antipsychotics are often regarded as the first line pharmacological approach for delirium.^14,15^ However, antipsychotics may have limited efficacy, and are not devoid of side effects (e.g. motor symptoms). Although controversial, electroconvulsive therapy is recognized as an efficient and safe way of treating delirium. It can be considered when agitation cannot be controlled with medication.^16^

In conclusion, prolonged delirium is not easy to detect or recognize. However, its early diagnosis and management in different scenarios are very important in order to improve patient prognosis.
